# The impact of stress on tournament entry

**DOI:** 10.1007/s10683-016-9496-x

**Published:** 2016-11-11

**Authors:** Thomas Buser, Anna Dreber, Johanna Mollerstrom

**Affiliations:** 1 School of Economics, University of Amsterdam, and Tinbergen Institute, Amsterdam, The Netherlands; 2 Department of Economics, Stockholm School of Economics, Stockholm, Sweden; 3 Department of Economics, Interdisciplinary Center for Economic Science (ICES), George Mason University, Research Institute for Industrial Economics, Stockholm, Sweden

**Keywords:** Competition, Gender differences, Experiment, Cortisol, Stress, C90, C91, J16, J71

## Abstract

Individual willingness to enter competitive environments predicts career choices and labor market outcomes. Meanwhile, many people experience competitive contexts as stressful. We use two laboratory experiments to investigate whether factors related to stress can help explain individual differences in tournament entry. Experiment 1 studies whether stress responses (measured as salivary cortisol) to taking part in a mandatory tournament predict individual willingness to participate in a voluntary tournament. We find that competing increases stress levels. This cortisol response does not predict tournament entry for men but is positively and significantly correlated with choosing to enter the tournament for women. In Experiment 2, we exogenously induce physiological stress using the cold-pressor task. We find a positive causal effect of stress on tournament entry for women but no effect for men. Finally, we show that although the effect of stress on tournament entry differs between the genders, stress reactions cannot explain the well-documented gender difference in willingness to compete.

**Electronic supplementary material:**

The online version of this article (doi:10.1007/s10683-016-9496-x) contains supplementary material, which is available to authorized users.

## Introduction

We ask whether stress reactions can explain individual differences in willingness to enter competitive environments. Experimental choices of competitive over non-competitive reward schemes have been shown to predict individual differences in career choices and labor market outcomes including labor market earnings (Buser et al. 2015; Reuben et al. 2015), choice of high-school study tracks (Buser et al. 2014), participation in a competitive college entrance exam (Zhang 2012), future salary expectations (Reuben et al. forthcoming) and investment decisions of entrepreneurs (Berge et al. 2015).

Using two laboratory experiments, we first examine whether participating in a tournament causes stress and whether differences in such stress reactions can explain willingness to enter a tournament at the individual level. We thereafter investigate whether there is a causal effect of exogenously induced stress on willingness to enter a tournament. Stress responses are typically triggered in situations that are novel, unpredictable, threatening or uncontrollable and it has been shown that situations where one’s performance is evaluated or compared to others’ are particularly stressing (Dickerson and Kemeny 2004; von Dawans et al. 2011). It hence seems likely that competing in a skills-based task induces stress for the average individual.

Our measure of individual willingness to choose a competitive reward scheme is based on the seminal study by Niederle and Vesterlund (2007). We follow their design and have participants perform a simple arithmetic task in three separate five-minute rounds. In the two initial rounds, participants are exposed to first an individual piece-rate payment scheme and then a competitive winner-takes-all tournament. Ahead of Round 3, they get to choose which of the two payment schemes to apply to that round. The decision of payment scheme in the third round is our measure of individual willingness to enter a tournament.

Our primary measure of acute stress comes from cortisol levels in saliva. Cortisol’s primary function is to mobilize glucose and it is considered the human body’s stress hormone. It reacts to both physical and psychological stressors through the autonomic nervous system and the hypothalamic–pituitary–adrenal axis (Dickerson and Kemeny 2004) and it can be easily and accurately measured in saliva (e.g., Vining et al. 1983; Kirschbaum and Hellhammer 1989). We also measure stress through self-assessments.

Our paper reports on the results of two experiments. Experiment 1 asks if an individual’s stress response to taking part in the mandatory tournament in Round 2 of the experiment predicts his or her willingness to enter the tournament in Round 3. Experiment 2 exogenously induces physiological stress through the commonly used cold-pressor task, where participants place their dominant hand in ice-cold water, and investigates if there is a causal effect of acute stress on tournament entry.

Starting with Niederle and Vesterlund (2007), a vast literature has documented that men with a given ability are more eager than equally able women to enter competitive environments (Croson and Gneezy 2009; Bertrand 2010; Niederle and Vesterlund 2010; Niederle 2014). This gender difference in willingness to compete can partly be traced to differences in confidence and risk aversion, but also when such factors are controlled for, an unexplained gender gap typically remains (Niederle and Vesterlund 2007; Buser et al. 2014).^1^ We relate this gender gap in willingness to compete to two other facts: first, competitions are often perceived as stressful, and second, research has documented that men and women sometimes react differently to acute stressors (this research has focused mostly, but not exclusively, on risk preferences, see e.g. Taylor et al. 2000; Lighthall et al. 2009; van den Bos et al. 2009; Angelucci and Córdova 2014; Kandasamy et al. 2014).^2^ Based on this literature, we investigate whether factors related to stress can explain the gender gap in willingness to compete.

We find that the mandatory tournament in Round 2 does increase stress levels relative to performing under the piece-rate scheme. We also find that for women, but not for men, stress reactions to the tournament are positively related to tournament entry. As expected, our randomized physiological stress treatment, the cold-pressor task, has a significant positive effect on cortisol levels. However, this increase in cortisol does not lead to a significant change in tournament entry for the sample as a whole. We do, however, find evidence of a positive effect of the treatment on women’s willingness to enter the tournament, indicating that the predictive power of cortisol reactions for women could be due to a causal effect of stress reactions on willingness to enter the tournament.

We replicate the common finding that women are less willing to compete than men conditional on performance, beliefs and risk attitudes. The positive relationship between cortisol levels and willingness to compete means that there is a large and statistically significant gender gap in choosing the tournament only for individuals with below-median cortisol reactions. The gender difference is small and insignificant for those with above-median reactions. Stress reactions cannot, however, explain a meaningful part of the aggregate gender gap in willingness to compete.

In Experiment 1, we also collect data on electrodermal activity measured through skin conductance. This is a proxy for both positive and negative psychological and physiological arousal. We test whether skin conductance changes or self-reported excitement in response to performing under individual and competitive incentives predict tournament entry in Round 3. We find that the responses to performing the task under the piece-rate and tournament schemes both positively predict tournament entry. This suggests that individuals who find the task more exciting are also more likely to choose to compete.

To our knowledge, our paper is the first to both explore the correlation between stress levels and tournament entry and provide a test of the causal impact of acute physiological stress on willingness to enter tournaments. We are also the first to provide a detailed investigation into the potential for stress to explain the gender gap in willingness to compete. We know of only three other studies that look at the correlation between stress and competitiveness. In a sample of male participants, Apicella et al. (2011) study willingness to compete in a maze task where participants have not performed any previous rounds of the task. Simply correlating baseline cortisol levels with competitiveness, they show that there is no significant relationship. Using the Niederle and Vesterlund (2007) competitiveness design, Buckert et al. (2015) correlate willingness to compete with heart rate variability and blood pressure, both which can be proxies of stress levels but also for other forms of arousal, as well as cortisol and testosterone in a mixed gender sample. They find no evidence of cortisol reacting to the forced competition or predicting willingness to compete. The study closest to ours is Zhong et al. (2015), who also use the Niederle and Vesterlund (2007) design in a mixed gender sample. They find that the cortisol response to performing in the task positively predicts tournament entry but find only a weak relationship between the cortisol response to the tournament and tournament entry once they control for the piece-rate response. They do not investigate gender differences in the link between cortisol and tournament entry.

Two other studies are more loosely related to our paper: Similar to Buckert et al. (2015), Halko et al. (2014) also use the Niederle and Vesterlund (2007) setup and explore to what extent heart rate variability correlates with gender differences in willingness to compete. They find that find men’s heart rate variability reacts more when competing than women’s. However, neither study finds that heart rate variability can explain the gender gap in competitiveness. In a different type of task, Goette et al. (2015) find that randomly stressing participants with a social stressor in a competitive context makes low-anxiety individuals overconfident whereas high-anxiety individuals become underconfident.^3^

The remainder of the paper is organized as follows: Sect. 2 describes the design of the two experiments, Sect. 3 presents our results, and Sect. 4 concludes.

## The two experiments

We conduct two laboratory experiments to examine the correlation between a person’s stress reaction to competing in a tournament and her willingness to voluntarily enter a tournament (Experiment 1), as well as investigate whether there is a causal effect of stress on the willingness to enter a tournament (Experiment 2).^4^

### Experiment 1: design

We closely follow the design of Niederle and Vesterlund (2007) and have participants perform a simple arithmetic task where they add sets of five two-digit numbers. They perform the task in three rounds, for 5 min per round. In Round 1, participants perform the task and are paid according to a piece-rate payment scheme where they get $1 per correctly solved problem. In Round 2, participants are placed in groups with three randomly chosen other participants. They perform the task again and the participant with the highest score in each group is paid $4 per correctly solved problem while the others receive nothing. In Round 3, participants choose between being paid according to a piece-rate payment scheme, as in Round 1, or according to a tournament payment scheme, as in Round 2. They thereafter perform the task again. If a participant chooses the tournament, she will compete against the second round performance of the same three participants (this guarantees that the choice has no externalities on the payoffs of others). Participants are not getting any feedback on their relative performance during the experiment. At the end they are paid in private for one randomly chosen round.

We measure cortisol levels from saliva at three different points during in the experiments. Saliva was sampled using oral swabs and samples were frozen immediately after collection and subsequently sent to the company Salimetrics where each sample was analyzed in duplicate. Cortisol levels are known to rise significantly within a few minutes of the onset of a stressor. After 10–20 min most of the effect has established itself and the effect peaks after 20–30 min (Dickerson and Kemeny 2004). The first saliva sample was collected at the start of the study (before any other instructions were given). The second saliva sample was collected 10 min after the end of Round 1 and the third sample 10 min after the end of Round 2. The 10 min delay after the end of the task implies that our measurement of cortisol was taken approximately 20 min after the onset of the stressor, i.e. after we started reading the instructions for the first and second task respectively.^5^ Thereby we get measures of the cortisol response from the piece-rate payment scheme in Round 1 and the mandatory tournament in Round 2 from the second and third saliva sample respectively.

We also measure self-reported emotions (stress, excitement, happiness and anger) before the start of the study, and immediately after Rounds 1 and 2. Immediately after Round 3 we also ask participants to guess their rank in Rounds 1 and 2 compared to the other three participants in their randomly assigned group. Participants receive $2 for each correct guess.

Skin conductance is regarded as a measure of electrodermal activity which is a proxy of psychological and physiological arousal (Mendes 2009). We measure it throughout Experiment 1 in order to be able to assess other types of arousal in addition to stress. We applied two electrodes on each participant’s non-dominant hand before the start of the experiment and used MindWare™ to measure the electrical conductance of the skin, which varies with its moisture level.

At the end of the study, participants answered a short questionnaire that among other things collected information on gender, age and the intake of hormonal medication, such as oral contraceptives. The questionnaire also elicited risk attitudes through a simple, non-incentivized question: “How do you see yourself: Are you generally a person who is fully prepared to take risks or do you try to avoid taking risks?”. The answer is on a scale from 0 (“unwilling to take risks”) to 10 (“fully prepared to take risks”). This question has been found to predict both incentivized choices in a lottery task and risky behaviors in different contexts in representative samples (Dohmen et al. 2011).^6^

### Experiment 2: design

The main difference between Experiment 1 and Experiment 2 is that in the latter we employ the cold-pressor task to exogenously increase cortisol levels in a random sample of participants. In the cold-pressor task, participants are asked to immerse their dominant hand into a bucket of ice-cold (0 °C) water for 90 s. The treatment is widely used as a physiological stressor as the painfulness of having the hand in the cold water typically produces a sharp increase in participants’ cortisol levels (see, e.g., Errico et al. 1993; Porcelli and Delagado 2009; Delaney et al. 2014).^7^

The cold-pressor task was administered to half of the participants immediately after Round 2. Randomization was done at the individual level. Participants in the control group had their dominant hand in a bucket of pleasantly warm (30–35 °C) water for 90 s, which has been found to not affect cortisol levels. This randomized treatment allows us to study the causal effect of an exogenous increase in cortisol on tournament entry.

As in Experiment 1, we collect saliva three times. We adjusted the waiting time for the third saliva measure so that it was taken 15 min after the end of the cold-pressor task, i.e. again close to 20 min after the onset of that particular stressor. Everything else regarding saliva measurements was done as in Experiment 1. The questionnaire for Experiment 2 was different from the one in Experiment 1 only in that it asked participants to report how hard they found it to keep the hand in the water, and to estimate how long they had the hand in the water (if they did not manage to do it for 90 s).

### Implementation

The experiments were conducted at the Harvard Decision Sciences Laboratory in Cambridge, MA. Experiment 1 was conducted in March and April 2014 and Experiment 2 in September and October 2014. Participants were recruited through the laboratory’s subject pool and mainly consisted of students at Harvard University. The experiments were programmed in z-Tree (Fischbacher 2007) and approved by the Harvard University Committee for the Use of Human Subjects.

When recruited, participants were instructed that they would not be allowed to eat, drink or do sports for at least an hour before the experiment (such activities are known to potentially influence cortisol measures). Participants were reminded about this on the day before the experiment, and upon arrival to the laboratory it was made sure by the experimenter that they had followed these instructions.

104 people participated in Experiment 1 (50 men and 54 women). Salimetrics was able to obtain reliable cortisol estimates for all three measurements for 101 participants which is the sample we use in our main analysis. Due to problems with the equipment, skin conductance measurements for all the relevant periods were obtained for 87 participants. 105 people participated in Experiment 2 (47 men, 58 women).^8^ Of these, we have to drop one participant who did not complete the entire experiment and one participant who did not use the saliva tube correctly. The number of people per session ranged from 4 to 10 in Experiment 1 and from 4 to 6 in Experiment 2. Recruitment was done in a way that ensured that the gender composition of each session was similar. Sessions in Experiment 1 lasted on average 65 min whereas sessions in Experiment 2 were on average 10 min longer. In both Experiment 1 and 2 participants earned on average $25.5.

Participants were seated in the lab one after the other, i.e. not simultaneously, and were seated in isolated cubicles in such a way that they could not see, or interact, with any other participant. It was also not possible for an individual participant to determine how many other participants were present in the laboratory at the same time. Sessions started at 1 pm and 3 pm for Experiment 1 and at 11am, 1:30 pm and 3:45 pm for Experiment 2. Cortisol has a natural diurnal pattern. We control for this by using standardized measures of cortisol as described below and by controlling for session dummies in all regressions.

Harvard undergraduates and undergraduates at other top universities are a very selective group which is not representative of the population in general or even the overall student population. However, we think that they are an especially interesting group to study because they make up the professional, academic and political elites of the future. Understanding what drives their willingness to compete, and the astonishing gender difference therein, is therefore especially relevant.

### Standardization of variables

Focusing on levels of salivary cortisol and skin conductance is not suitable for our analysis given that levels vary strongly between individuals in ways that are unrelated to the experiment (cortisol levels in saliva depend for example on how much an individual salivates, while skin conductance levels depend on a person’s natural tendency to sweat).

We therefore use individual baseline levels to standardize the measurements taken during the experiment. That is, we divide the second and third measures of cortisol and mean skin conductance levels during the task performances by the measurements taken at the start of the experiment. Baseline skin conductance levels are defined as the average level of skin conductance during the period between reading the welcome screen and reading the instructions for the first round (this is also the period during which the first saliva sample was collected). Thus for skin conductance we focus on skin conductance response rather than skin conductance levels (see Mendes 2009 for a discussion of the difference between these measures). All results reported in the paper regarding cortisol and skin conductance refer to these standardized measures.

## Results

Table 1 shows descriptive statistics from the two experiments. The averages of the experimental variables are similar across experiments. Participants solved approximately 10 problems in Round 1 and approximately 11 problems in Round 2. They are overconfident on average with a mean guessed rank of approximately 2 for the tournament (the true average rank is 2.5). 40 percent of participants in Experiment 1 and 43 percent in Experiment 2 chose the tournament over the piece rate. The table also confirms that the randomization in Experiment 2 was successful: there are no significant differences in performance, confidence or risk attitudes between the treatment and control groups. The table also contains means of the standardized cortisol and skin conductance measurements, which are discussed below.

**Table 1:** Summary statistics

	Experiment 1	Experiment 2			
		All	Control	Treatment	p
Performance round 1	9.90	10.19	10.24	10.15	0.91
Performance round 2	11.67	11.38	11.34	11.42	0.93
Guessed rank round 1	2.16	2.27	2.14	2.40	0.13
Guessed rank round 2	1.94	1.98	1.90	2.06	0.37
Risk attitudes	5.59	5.85	5.64	6.06	0.39
Choosing competition	0.40	0.43	0.40	0.45	0.59
Standardized cortisol (after PR)	1.04	1.02	1.04	1.00	0.62
Standardized cortisol (after competition/treatment)	1.13	1.21	0.98	1.43	0.01
Standardized skin conductance during PR	1.26				
Standardized skin conductance during competition	1.35				
Self-rated stress (baseline)	5.24	5.17	5.14	5.21	0.88
Self-rated stress (after PR)	5.24	5.53	5.22	5.83	0.19
Self-rated stress (after competition)	5.8	6.17	6.00	6.32	0.50
Self-rated excitement (baseline)	5.11	5.70	5.58	5.81	0.57
Self-rated excitement (after PR)	5.87	6.32	6.16	6.47	0.48
Self-rated excitement (after competition)	6.08	6.33	6.58	6.09	0.30

p-values are from t-tests. Standardized cortisol levels means cortisol divided by baseline values. Standardized skin conductance means mean skin conductance during task performance divided by mean baseline levels. Self-rated stress and self-rated excitement are on a scale from 1 to 10

### Does competing cause stress?

**Fig. 1:**
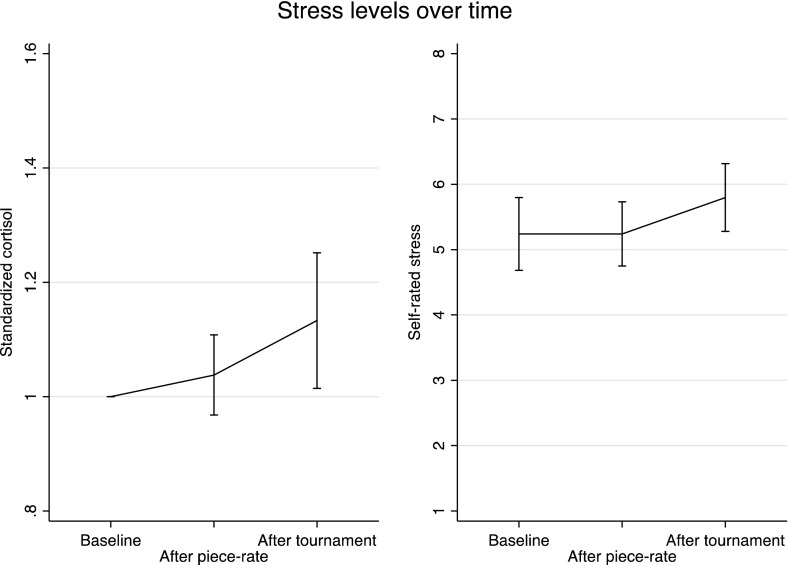
Stress levels, Experiment 1. *Error bars* represent 95 % confidence intervals

Figure 1 shows standardized cortisol levels and self-rated stress levels after the piece-rate round (Round 1) and after the tournament round (Round 2) in Experiment 1. We find that competition causes stress: cortisol levels after the piece-rate performance are not significantly different from baseline levels (an increase of 3.8 percent, p = 0.29; paired *t* test) but levels after the competition are significantly higher compared to baseline (an increase of 13.3 percent, p = 0.03) and compared to after the piece-rate round (an increase of 11.6 percent, p = 0.02). In a similar vein, we find that self-rated stress levels after the piece-rate round are not higher than baseline levels (p = 1.00) while competition levels are 10.6 percent higher than baseline (p = 0.02) and piece-rate levels (p < 0.01).^9^

**Fig. 2:**
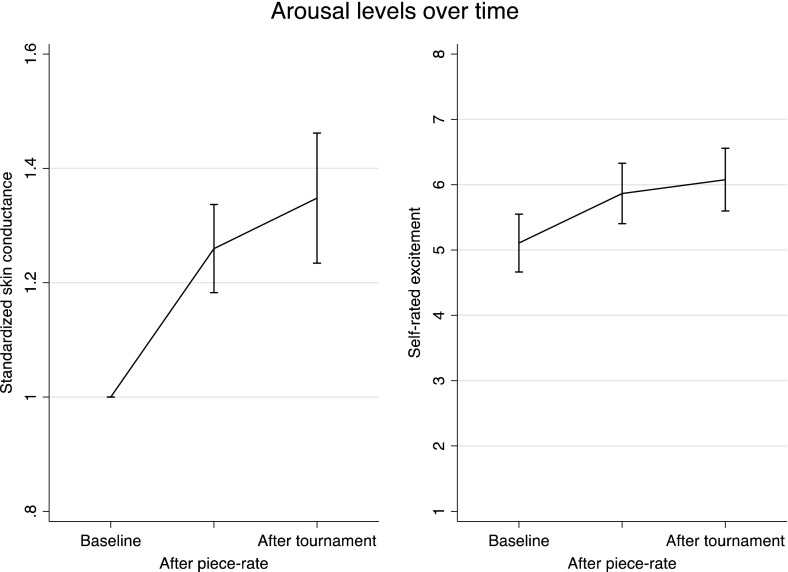
Arousal levels, Experiment 1. *Error bars* represent 95 % confidence intervals

We also analyze skin conductance response and self-reported excitement as two proxies of arousal. The piece-rate and tournament response of skin conductance are defined as average skin conductance during the five-minute piece-rate and tournament performances (rounds one and two respectively), standardized by baseline levels. Figure 2 suggests that performing the task itself causes arousal. Compared to baseline, piece-rate skin conductance is 26 percent higher (p < 0.01) and self-rated excitement similarly increases by 15 percent compared to the baseline (p < 0.01). Skin conductance levels increase 8 percent during the competition compared to piece-rate levels (p < 0.01) and 35 percent compared to baseline (p < 0.01). Self-rated excitement increases by 4 percent during the competition compared to piece-rate levels (p = 0.23) and 19 percent compared to baseline (p < 0.01).

### Can differential stress reactions explain individual differences in tournament entry?

**Fig. 3:**
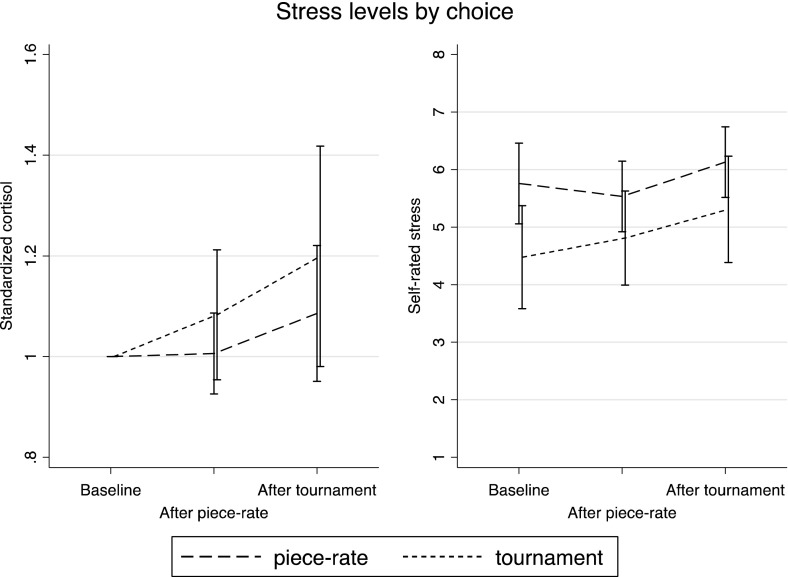
Stress levels by choice, Experiment 1. *Error bars* represent 95 % confidence intervals

We next explore whether stress reactions to competing in Round 2 predict willingness to enter the tournament in Round 3. Figure 3 illustrates that neither the change in standardized cortisol nor the change in self-rated stress in response to competing differ much between those participants who chose the piece-rate and those who chose the competition in Round 3.

**Fig. 4:**
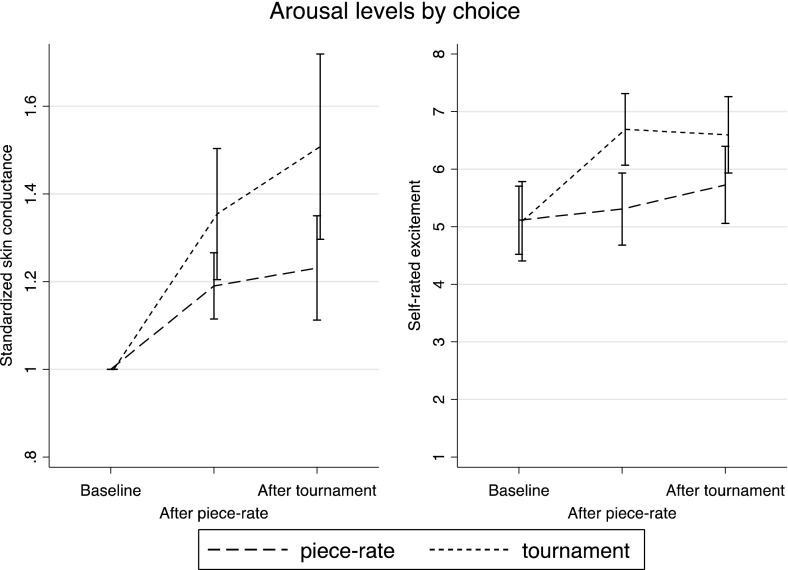
Arousal levels by choice, Experiment 1. *Error bars* represent 95 % confidence intervals

These results are confirmed by the probit results in Table 2, where we regress a tournament entry dummy on cortisol responses and self-rated stress responses. From Fig. 4, we can see that those who choose to compete in Round 3 experience a somewhat higher increase in cortisol and self-rated stress during the piece-rate performance. However, columns 1 and 2 of Table 2 show that this difference is not significant. That is, how stressed a participant gets by performing the task under piece-rate incentives does not predict choosing the tournament. Furthermore, neither the cortisol reaction to competing (Column 3) nor the reaction of self-rated stress (Column 4) predict the choice of payment scheme in Round 3. Apart from being statistically insignificant, the magnitudes of the coefficients are small. As an illustration, using the coefficient from Column 3 the average cortisol reaction would lead to an increase in the likelihood of choosing the tournament in Round 3 of around 1 percentage point.

**Table 2:** Tournament entry and stress (marginal effects from probit), Experiment 1

	(1)	(2)	(3)	(4)	(5)	(6)	(7)
Standardized cortisol after PR	0.143				0.150		0.117
	(0.138)				(0.223)		(0.222)
Standardized stress after PR		0.073				0.104*	0.091
		(0.048)				(0.062)	(0.057)
Standardized cortisol after comp.			0.062		−0.006		−0.007
			(0.083)		(0.133)		(0.131)
Standardized stress after comp.				0.025		−0.034	−0.029
				(0.044)		(0.051)	(0.049)
N	101	101	101	101	101	101	101
Pseudo-R^2^	0.073	0.080	0.070	0.068	0.074	0.082	0.086

Dependent variable: dummy indicating choice of competition in Round 3. All regressions control for session dummies. Robust standard errors in parentheses

*** p < 0.01; ** p < 0.05; * p < 0.1

In Fig. 4 and Table 3, we explore whether changes in arousal and excitement predict tournament entry in Round 3. As Fig. 4 shows, those participants who choose to compete in Round 3 show a stronger skin conductance response both when comparing the piece-rate performance to baseline (p = 0.04; paired t-test) and when comparing the tournament to the piece rate (p = 0.06). Those who choose to compete also show a significantly stronger reaction in self-rated excitement during the piece-rate performance (p < 0.01) but not during the tournament compared to piece rate (p = 0.15).

**Table 3:** Tournament entry and arousal (marginal effects from probit), Experiment 1

	(1)	(2)	(3)	(4)	(5)	(6)	(7)
Standardized skin cond. PR	0.319**				0.040		0.026
	(0.143)				(0.316)		(0.268)
Standardized excitement PR		0.367***				0.454***	0.461***
		(0.081)				(0.133)	(0.138)
Standardized skin cond. comp.			0.224**		0.199		0.189
			(0.091)		(0.212)		(0.179)
Standardized excitement comp				0.185***		−0.082	−0.083
				(0.066)		(0.103)	(0.109)
N	87	87	87	87	87	87	87
Pseudo-R^2^	0.106	0.195	0.111	0.126	0.111	0.199	0.248

Dependent variable: dummy indicating choice of competition in Round 3. All regressions control for session dummies. Robust standard errors in parentheses

The number of observations is slightly smaller than in Table 3 because for some participants, skin conductance was not measured at the relevant moments due to equipment malfunctioning

*** p < 0.01; ** p < 0.05; * p < 0.1

This is further explored in Table 3, where we regress a dummy for choosing the competition in Round 3 on skin conductance responses and responses in self-rated excitement. Columns 1 to 4 confirm that participants who experienced an increase in arousal in response to performing in the task are more likely to choose to compete. When we add both the piece-rate and the tournament skin-conductance responses in Column 5, the coefficient on the second is much larger, indicating that it is people who get excited by competing in a tournament who are more likely to enter the tournament. However, the opposite is the case for self-rated excitement (Column 6). When all measures of arousal (piece-rate and competition skin conductance responses and self-rated excitement) are included in Column 7, although most measures are not individually significant, they jointly significantly predict tournament entry (p = 0.000; Wald test).

Given that some studies have found gender differences in the behavioral consequences of stress responses, we will now investigate whether our null result for cortisol hides a differential response of men and women to the stress reaction caused by the tournament. In Column 1 of Table 4, we regress the choice of compensation scheme in Round 3 on standardized cortisol levels after the piece-rate performance in Round 1, a female dummy and the interaction of the two. In Column 2, we do the same for cortisol levels after the competition in Round 2. In Column 3, we add both cortisol measures and their interactions with gender. In all regressions, cortisol responses were normalized to have mean zero so that the coefficient on the female dummy represents the gender difference for participants with an average cortisol response. Below the regressions, we report p-values from Wald tests for the effect of the cortisol responses on tournament entry for women.

**Table 4:** Tournament entry and stress: gender differences (marginal effects from probit), Experiment 1

	(1)	(2)	(3)	(4)	(5)
	All	All	All	Cortisol > median	Cortisol < median
Female	−0.252***	−0.252***	−0.253***	−0.139	−0.343***
	(0.081)	(0.082)	(0.081)	(0.134)	(0.101)
Standardized cortisol after PR	−0.077		0.055		
	(0.164)		(0.282)		
Female*Std. cortisol after PR	0.549**		0.239		
	(0.229)		(0.363)		
Standardized cortisol after comp.		−0.072	−0.094		
		(0.097)	(0.164)		
Female*Std. cortisol after comp.		0.369**	0.256		
		(0.145)	(0.227)		
p value cortisol effect for women (PR)	0.009		0.223		
p value cortisol effect for women (comp.)		0.015	0.330		
Joint p value			0.028		
N	101	101	101	51	50
Pseudo-R^2^	0.160	0.161	0.168	0.015	0.109

Dependent variable: dummy indicating choice of competition in Round 3. All regressions control for session dummies with the exception of Columns 4 and 5. p values for the cortisol effect for the female subsample are from post-estimation Wald tests. Robust standard errors in parentheses

*** p < 0.01; ** p < 0.05; * p < 0.1

While the effect of the standardized cortisol measures is close to zero for the male subsample, the gender interactions in both Column 1 and Column 2 are large and statistically significant. Women’s reaction to the cortisol responses is positive and significant in both cases. When we add both measures in Column 3, the coefficients on both gender interactions are of similar magnitude but estimates are noisier and not statistically significant. The effect of the two cortisol measurements is jointly significant for the female subsample. In Columns 4 and 5, we split the sample into those whose standardized cortisol levels after the tournament in Round 2 are below and above the median. While there is a strong and significant gender gap in the likelihood of choosing the tournament for those with a below-median cortisol response, the gender difference is much smaller for those with an above-median response.

In summary, we find that for men the willingness to enter a tournament does not depend on stress reactions: those who show a strong cortisol increase when competing are just as likely to choose the tournament as those who show a weak response. For women on the other hand, those who experience a strong cortisol response are more likely to choose the tournament and this effect is strong enough that there is no significant gender gap in entry for participants with an above-median standardized cortisol response to competition. We will discuss the gender gap in tournament entry, and how it relates to stress reactions, in more detail in Sect. 3.4.

### Is there a causal effect of stress on tournament entry?

We now move on to investigate whether there is a causal effect of stress on tournament entry. This will also help us disentangle possible explanations for the gender difference in the predictive power of cortisol responses: is it simply the case that women who show a stronger cortisol response have a preference for competition, or does a stronger stress response actually make women compete more?

**Fig. 5:**
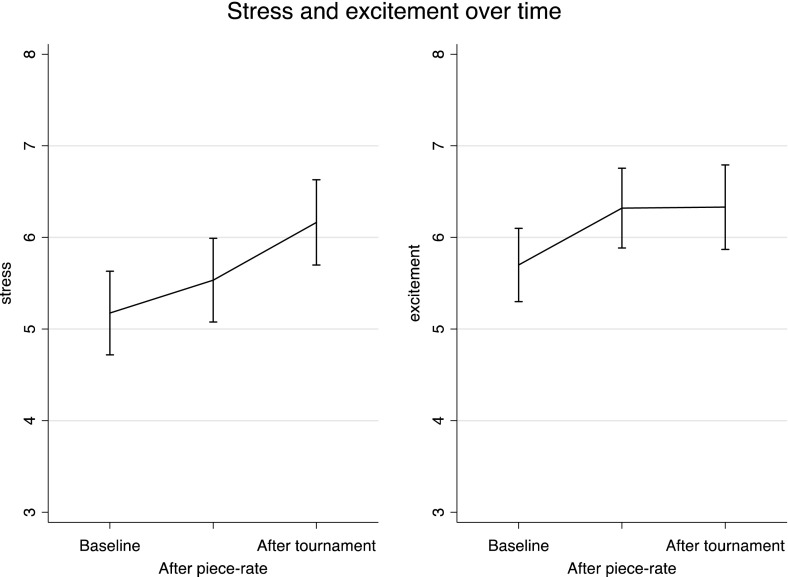
Self-rated stress and excitement, Experiment 2. *Error bars* represent 95 % confidence intervals

Given that our third cortisol measurement was taken after the cold-pressor task in Experiment 2 (to check that the manipulation worked) we cannot replicate the cortisol analysis from Experiment 1. However, we measured self-rated stress and excitement and these measurements were taken before the stress treatment. As Fig. 5 shows, we replicate the effects of competition found in the first experiment: self-rated stress increases by 11.4 percent in response to competing (p < 0.01; paired t-test) while there is no change in excitement (p = 0.96). As in Experiment 1, excitement increases in response to the piece-rate performance (p < 0.01). Contrary to Experiment 1, there is now also an increase in self-rated stress in response to the piece-rate performance (p = 0.06).

**Fig. 6:**
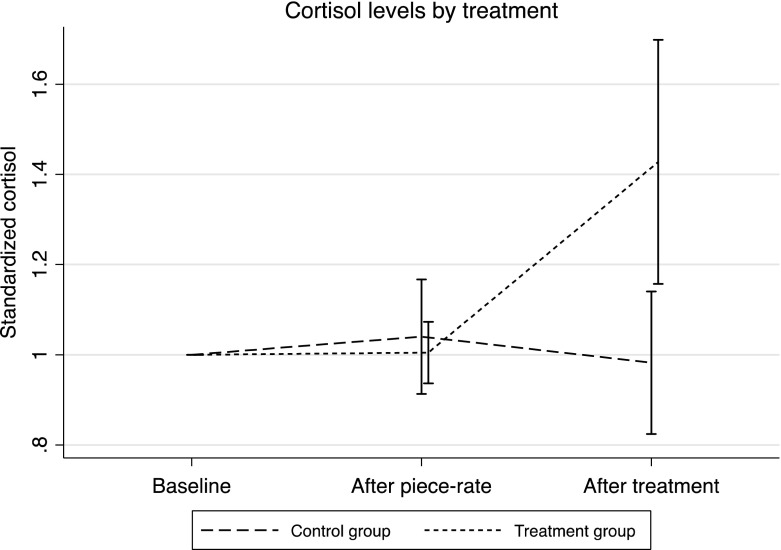
Cortisol levels by stress treatment, Experiment 2. *Error bars* represent 95 % confidence intervals

Figure 6 shows that the stress treatment was successful, increasing cortisol levels by 44 percent (p < 0.01) relative to the control group. However, there is no significant difference in tournament entry between the treatment and control groups (45 percent of the treatment and 40 percent of the control group chose the tournament over the piece rate; p = 0.59, Chi squared test). This result is confirmed by Column 1 of Table 5, where we regress tournament entry on a treatment dummy.^10^

**Table 5:** Causal effect of stress on willingness to compete: gender differences (probit), Experiment 2

	(1)	(2)	(3)	(4)	(5)	(6)	(7)	(8)	(9)
	All	All	Treatment hard	Treatment effect	All	All	All	Treatment	Control
Cold water	0.073	−0.122	-0.246*	−0.213					
	(0.089)	(0.126)	(0.133)	(0.142)					
Female		−0.493***	−0.555***	−0.515***	−0.303***	−0.306***	−0.309***	−0.160	−0.375***
		(0.105)	(0.106)	(0.109)	(0.078)	(0.072)	(0.073)	(0.131)	(0.087)
Female*cold water		0.351**	0.562***	0.493**					
		(0.169)	(0.193)	(0.209)					
Standardized cortisol (2nd)					−0.071		0.132		
					(0.133)		(0.161)		
Female* Std. cortisol (2nd)					0.443		−0.555		
					(0.295)		(0.421)		
Standardized cortisol (3rd)						−0.105*	−0.150*		
						(0.058)	(0.082)		
Female* Std. cortisol (3rd)						0.347***	0.517***		
						(0.108)	(0.156)		
p val cold water effect for women		0.041	0.023	0.063					
p value cortisol effect for women (PR)					0.170		0.281		
p value cortisol effect for women (comp.)						0.017	0.010		
Joint p value							0.018		
N	103	103	78	81	103	103	103	53	50
Pseudo-R^2^	0.099	0.208	0.246	0.183	0.195	0.240	0.250	0.019	0.139

Dependent variable: dummy indicating choice of competition in Round 3. Cold Water is a dummy indicating a participant has been randomly assigned to the treatment group. All regressions control for session dummies with the exception of Columns 8 and 9. Robust standard errors in parentheses

*** p < 0.01; ** p < 0.05; * p < 0.1

Following our finding of a gender difference in the impact of cortisol in Experiment 1, we will now analyze whether there is a gender difference in the impact of the stress treatment. In Column 2 of Table 5, we interact the treatment dummy with gender. The effect of the treatment for men is negative and sizeable at 12 percentage points but not statistically significant. On the other hand, the treatment effect for women is large, positive and statistically significant at the 5-percent level. In Columns 3 and 4, we repeat this analysis limiting the treatment group to participants for whom the treatment was effective. To check whether the stress treatment worked, we asked the following question in the post-experimental questionnaire: “Did you find it hard or easy to keep the hand in the water for 90 s?” (on a scale from 1 = “very hard” to 10 = “very easy”). In Column 3, we restrict the treatment group to those who gave an answer of 3 or lower. The coefficient on the gender interaction is even larger while the negative effect for the male subsample is also larger and statistically significant at the 10 % level. In Column 4, we restrict the treatment group to those whose post-treatment cortisol levels are above the median, basically restricting the sample to the control group and those for whom the treatment had a noticeable effect on their cortisol levels. The results are very similar.

In Columns 5 to 7, we replicate the analysis in Table 4, using standardized cortisol levels at the second and third measurements (that is, after the piece-rate round and after the stress treatment) instead of a treatment dummy. The result is the same as in the first experiment: the impact of the cortisol response to competing on tournament entry is small and negative for men but large, positive and significant for women. Column 7 shows that the impact of post-treatment cortisol levels is significantly negative for men and significantly positive for women when controlling for the post piece-rate measurement.^11^ Finally, in Columns 8 and 9, we run separate regressions for the treatment and control groups. While we find a large and statistically significant gender difference in the control group, the gender gap is much smaller and not statistically significant in the treatment group.

Together, the results of the two experiments indicate that tournament entry for women, but not men, is partially explained by cortisol reactions. The results of the second experiment suggest that this is at least partially due to a causal effect (higher cortisol levels leading to a higher willingness to enter the tournament for women).

### Can stress reactions explain the gender difference in willingness to compete?

A large literature demonstrates that women are less willing to compete than men (where willingness to compete is defined as tournament entry controlling for performance and, sometimes, beliefs and risk preferences). In this section we will first demonstrate that our data replicates this pattern. Given that we find a gender difference in the effect of stress reactions on tournament entry, we will then investigate whether stress reactions can help explain the gender gap in willingness to compete.

In Table 6, we show summary statistics by gender. Women are significantly less likely to choose to compete in Round 3 than men in both experiments. Even though there are no significant gender differences in performance, in Experiment 1 only 28 percent of the women choose to compete while the corresponding number for men is 52 percent. In Experiment 2, 30 percent of women and 59 percent of men compete.

**Table 6:** Summary statistics by gender

	Experiment 1			Experiment 2		
	Men	Women	p	Men	Women	p
Performance round 1	10.31	9.46	0.24	10.33	10.09	0.76
Performance round 2	11.87	11.46	0.62	11.89	10.96	0.31
Guessed rank round 1	2.06	2.28	0.22	2.28	2.26	0.91
Guessed rank round 2	1.96	1.92	0.81	2.00	1.96	0.84
Risk attitudes	6.41	4.70	0.00	6.26	5.53	0.13
Choosing competition	0.52	0.28	0.01	0.59	0.30	0.00
Standardized cortisol (after PR)	1.05	1.03	0.79	1.11	0.95	0.03
Standardized cortisol (after treatment)	1.15	1.12	0.79	1.38	1.07	0.06
Standardized skin conductance during PR	1.21	1.31	0.18			
Standardized skin conductance during competition	1.33	1.37	0.73			
Self-rated stress (baseline)	4.57	5.96	0.01	4.98	5.33	0.45
Self-rated stress (after PR)	4.67	5.86	0.02	5.39	5.65	0.58
Self-rated stress (after competition)	5.17	6.48	0.01	5.80	6.46	0.17
Self-rated excitement (baseline)	5.67	4.50	0.01	5.72	5.68	0.94
Self-rated excitement (after PR)	6.39	5.30	0.02	6.35	6.30	0.91
Self-rated excitement (after competition)	6.17	5.98	0.70	6.48	6.21	0.57

p-values are from t-tests. Standardized cortisol levels means cortisol divided by baseline values. Self-rated stress and self-rated excitement are on a scale from 1 to 10

In Table 7, we use probit regressions to estimate the effect of stress reactions on the gender difference in willingness to compete. Because we found the same gender difference in the effect of stress reactions on tournament entry in both experiments, we pool the data from both experiments to increase precision. In Column 1, we regress a tournament entry dummy on a gender dummy. Over both experiments, women are 26 percentage points less likely to choose the tournament. In Column 2, we additionally control for performance in Rounds 1 and 2, guessed rank in Rounds 1 and 2 and the questionnaire measure of risk preferences. The coefficient on the gender dummy is still 20 percentage points. Given that our controls are surely measured with error, this remaining gender gap is likely an overestimate (for example, Gillen et al. 2015 find that confidence and risk aversion can explain most of the gender gap when multiple and more accurate controls are used; see also van Veldhuizen 2016). Nevertheless, the regression results confirm that the women in our sample are significantly less willing to compete than the men.

**Table 7:** Stress and the gender gap in willingness to compete (marginal effects from probit): pooled data

	(1)	(2)	(3)	(4)	(5)	(6)	(7)	(8)
	All	All	All	All	All	All	Cortisol > median	Cortisol < median
Female	−0.259***	−0.204***	−0.198***	−0.197***	−0.190***	−0.189***	−0.115	−0.240***
	(0.058)	(0.051)	(0.051)	(0.051)	(0.051)	(0.051)	(0.078)	(0.067)
Standardized cortisol (2nd)			0.064	0.031	−0.020	0.014		
			(0.070)	(0.099)	(0.086)	(0.113)		
Standardized cortisol (3rd)				0.023		−0.020		
				(0.056)		(0.054)		
Female*Std. cortisol (2nd)					0.333**	0.032		
					(0.134)	(0.191)		
Female*Std. cortisol (3rd)						0.197**		
						(0.086)		
p value cortisol effect for women (PR)					0.004	0.762		
p value cortisol effect for women (comp.)						0.011		
Joint p value						0.001		
Scores, beliefs and risk		√	√	√	√	√	√	√
N	204	204	204	204	204	204	102	102
Pseudo-R^2^	0.109	0.376	0.378	0.379	0.393	0.407	0.312	0.357

Dependent variable: dummy indicating choice of competition in Round 3. All regressions except Columns 7 and 8 control for session dummies. Robust standard errors in parentheses

*** p < 0.01; ** p < 0.05; * p < 0.1

In Columns 3 and 4 we add standardized cortisol at the second and third measurements. The coefficient on the female dummy hardly changes, showing that although competing is stressful and women react differently to stress than men, this has no impact on the aggregate gender gap in willingness to compete. In Columns 5 and 6, we add interactions between the standardized cortisol measures and gender. When we add both measures in Column 6, the interaction of gender with the post-competition measure is large and significant while the effect is close to zero for men, confirming that the effect of stress on willingness to compete is positive and significant for women only.

The results of these regressions demonstrate why controlling for stress reactions does not change the gender gap. The coefficient on the female dummy is equal to 19 percentage points. This means that women with an average stress reaction are 19 percentage points less likely to enter the competition than men. The coefficient on the interaction is equal to 20 percentage points. This means that a woman with a stress reaction that is one standard deviation above the average is only 5 percentage points less willing to compete than the average man while a woman with a stress reaction that is one standard deviation below the average is 33 percentage points less willing to compete. In Columns 7 and 8, we show that, due to the effect of cortisol on tournament entry for women, there is a large and significant gender gap in willingness to compete only in the subsample of participants with below-median post-competition (and, in case of Experiment 2, post-treatment) cortisol levels.

### Is there an effect of stress on performance?

Some studies find that (acute) stress is good for productivity and performance (e.g., Kavanagh 2005). We will therefore briefly discuss whether, in our experiments, stress reactions and randomly induced stress have an impact on performance. Table 8 shows OLS regressions of scores in Rounds 2 and 3 on stress indicators controlling for scores in Round 1. The coefficients on the stress indicators are therefore conditional on Round 1 performance; that is, they show whether stress reactions correlate with or cause an increase in performance. We start with the first experiment, with focus on Round 2 performance since in this round all participants had to compete. In Columns 1 and 2, we regress scores in Round 2 on standardized cortisol after the piece-rate performance and after the tournament performance. In both cases, the coefficient is statistically insignificant. When we add both measures simultaneously in Column 3, they are individually and jointly insignificant. That is, we find no evidence for a correlation between stress reactions and performance. In Columns 4 to 7, we move to the second experiment to determine whether there is a causal effect of stress on performance in Round 3. In Column 4, we show that the random stress treatment has no impact on scores in Round 3 conditional on initial scores and the choice of payment scheme. In the last three columns we show that standardized cortisol levels after the piece-rate performance (before the random stress treatment) and after competition (after the random stress treatment) do not predict Round 3 scores either.

**Table 8:** Cortisol and performance

Dependent variable	(1)	(2)	(3)	(4)	(5)	(6)	(7)
	Experiment 1			Experiment 2			
	Score round 2	Score round 2	Score round 2	Score round 3	Score round 3	Score round 3	Score round 3
Std. cortisol after PR	0.695		−0.004		−0.071		−0.090
	(0.943)		(1.111)		(1.123)		(1.564)
Std. cortisol after competition		0.541	0.543			−0.011	0.012
		(0.604)	(0.802)			(0.325)	(0.483)
Cold Water				0.044			
				(0.508)			
Score round 1	0.852***	0.839***	0.838***	0.991***	0.992***	0.991***	0.992***
	(0.085)	(0.088)	(0.089)	(0.078)	(0.083)	(0.080)	(0.083)
Competes				1.335**	1.340*	1.340**	1.340*
				(0.658)	(0.674)	(0.669)	(0.674)
Joint p value			0.674				0.998
N	101	101	101	103	103	103	103
R^2^	0.674	0.676	0.676	0.765	0.765	0.765	0.765

Cold Water is a dummy indicating a participant has been randomly assigned to the treatment group. All regressions control for session dummies. Robust standard errors in parentheses

*** p < 0.01; ** p < 0.05; * p < 0.1

## Discussion

We document that performing under competitive incentives is stressful for the average individual. We find no impact of stress on tournament entry for men. Men who show a stronger stress reaction to performing the task either under the piece-rate or the competitive incentive scheme are neither more nor less likely to enter the tournament and our randomized exogenous stress treatment has no impact on men’s tournament entry. However, stress reactions to performing under competitive incentives positively predict tournament entry for women. This correlation is strong enough that the gender gap in tournament entry is substantially smaller for those participants with a high stress reaction to competition. We investigate whether this is due to selection (women who show a stronger cortisol reaction to performing in a competition liking competition more) or to a causal effect of stress on tournament entry. We find evidence that the randomized exogenous stress treatment has a large and positive effect on women’s willingness to enter tournaments, implying a causal relationship.

In order to design and implement adequate policies that address gender differences in labor market outcomes, it is important to know the mechanisms underlying the gender gap in willingness to compete. To what extent do our results indicate that stress reactions can “explain” the gender gap in competitiveness? We find that for women, willingness to compete is more sensitive to cortisol changes than for men. While this furthers our understanding of *individual* willingness to enter tournaments, it cannot explain the *aggregate* gender gap in tournament entry: controlling for cortisol reactions has no impact on the overall gender gap. However, our results provide the new insight that women who have a strong cortisol reaction to competing are almost as likely as men to enter the tournament, while women with a weak cortisol reaction are even less likely to choose the tournament than the average woman.

Cortisol prepares the individual for an oncoming confrontation or fight and it therefore makes sense that those who experience a stronger cortisol reaction are more willing to face a competition. The question is why we see evidence of this only for women. Potentially, we do not find this effect for men because of their already very high willingness to compete (many men enter the tournament in Round 3 with very low chances to win). It is therefore possible that whereas men compete no matter what in this type of setting, for women stress reactions help to overcome an inclination to avoid competitive situations.

While acute stress reactions can be beneficial, chronic stress is associated with a long list of adverse health outcomes. If our result that competitive payment schemes lead to increased stress for the average individual extrapolates to the workplace, it could mean that competitive remuneration schemes and promotion mechanisms have potential implications for long-term health outcomes of employees. This, in turn, opens up an interesting avenue for research into not only the performance effects but also the health consequences of different payment schemes and other workplace practices.^12^

## Electronic supplementary material

Below is the link to the electronic supplementary material.

Supplementary material 1 (DOC 30 kb)

## Footnotes

1 The fact that women to a significantly higher extent than men opt out of this type of competitions has been documented in many (but not all) societies and across age groups (e.g., Gneezy et al. 2009; Cárdenas et al. 2012; Flory et al. 2012; Mayr et al. 2012; Andersen et al. 2013; Almås et al. 2015; Apicella and Dreber 2015; Sutter and Glätzle-Rützler 2015), and this tendency appears to be particularly strong for math-related tasks (Günther et al. 2010; Grosse and Reiner 2010; Dreber et al. 2014). See Flory et al. (2010) for a field experiment in a real labor market.

2 There is also a literature investigating the links between stress and preferences (again mostly risk-taking) without investigating potential gender differences, and with mixed results. See, e.g., Coates and Herbert (2008), Porcelli and Delgado (2009), von Dawans et al. (2012), Chumbley et al. (2014) and Schipper (2014). Moreover, there is a vast literature correlating stress indicators such as cortisol levels with other types of risk taking than economic risk taking; see for example Mehta et al. (2015).

3 The competitive context involves participants making a decision based on self-confidence about their cognitive ability.

4 All experimental instructions and questionnaires are available in the online appendix.

5 Participants were told to wait and not do anything during the 10 min delay. The experimenters made sure that these instructions were followed and there were no issues with non-compliance.

6 The relationship between risk preferences and stress is not the focus of our study as this has been explored extensively in past work with incentivized measures. We include this non-incentivized measure in order to use it as a control variable.

7 Even though the majority of published results show an effect of the cold-pressor task on cortisol levels, there are also examples of null effects. Thus we look at cortisol levels to make sure the manipulation worked.

8 The reason that the sample size is not divisible by 4 is that even though participants were randomly grouped with three other participants, these participants were not necessarily grouped with the same other participants.

9 The order of the piece-rate and competition performances was not randomized which could lead to spillovers of cortisol from round 1 to the measured cortisol levels after round 2, since although cortisol levels peak quickly they can stay elevated for up to an hour after the onset of the stressor (Dickerson and Kemeny 2004). However, we take our post piece-rate measurement near the peak. When we measure cortisol levels post competition, the peak of the response to the piece-rate has consequently passed. Any additional increase in cortisol between the two measurements is therefore due to the competition round. This means that the difference between the competition and the baseline measurements is an upper bound for the effect of competition on cortisol and the difference between the competition and piece-rate measurements is a lower bound. Moreover, the fact that neither cortisol nor self-assessed stress increased significantly during the first round makes it unlikely that our results are reflecting a mere order effect.

10 Kirschbaum et al. (1994) find that oral contraceptives might dampen the cortisol response to stress and the results of Buser (2012) raise the possibility of a direct impact of oral contraceptives on the willingness to compete. When we control for contraceptive intake as a robustness check, the results do not change. Controlling for the intake of psychoactive medicine does not change the results either.

11 Note that, contrary to the regressions in Columns 2 to 4, these estimates do not necessarily represent a causal effect of stress on tournament entry. An alternative explanation for these results is that women who have a higher reactivity to the cold-pressor treatment are more inclined to choose the tournament for other reasons.

12 One study which directly focuses on this topic is Falk et al. (2011) who find a link between the perceived unfairness of the remuneration for a tedious task and heart rate variability, which predicts heart diseases in the long run.
