# Supplementary material for: The impact of stress on tournament entry
Source: Exp Econ. 2016 Nov 11;20(2):506–30. doi: 10.1007/s10683-016-9496-x (PMC5425495; doi:10.1007/s10683-016-9496-x)
Supplement: Supplementary file 1 — Supplementary material 1 (DOC 30 kb) [file 10683_2016_9496_MOESM1_ESM.doc]

Hi and welcome! In this experiment you will be asked to complete different tasks. We will also ask you for three samples of saliva. Please press OK to get started with the experiment.

OK

Please describe your feelings at this moment. [Four 1-10 scales with agree/disagree for feeling happy, angry, stressed and excited respectively.]

OK

We now ask you to provide the first saliva sample. 1. Remove the top cap of the tube to expose the round swab. Do not remove the holder that the swab is sitting in. 2. Place the swab directly into your mouth by tipping the tube so the swab falls into your mouth. Do not touch the swab with your fingers. 3. Keep the swab under the front of your tongue for 100 seconds. 4. Spit the swab back into the tube. Do not touch the swab with your fingers. 5. Replace the cap. Make sure cap is on tightly.6. Press the OK button after one of us has collected the tube. To help you keep track of the 100 seconds that you will have the swab in your mouth a clock (which counts seconds) is displayed below. Please note the time when you place the swab in your mouth. Spit out the swab into the tube after 100 seconds. If you have any questions, please raise your hand or press the help button.

OK

In this experiment you will be asked to complete three different tasks. None of these will take more than 5 minutes. At the end of the experiment we will randomly select one of the tasks. This is the task that will be relevant for your profit. Once you have completed the three tasks we determine which task counts for your profit by randomly drawing a number between 1 and 3. The method we use to determine your earnings varies across tasks. Before each task we will describe in detail how your payment is determined.

OK

Instructions for task 1. For Task 1 you will be asked to calculate the sum of five randomly chosen two-digit numbers. You will be given 5 minutes to do a series of these problems. You are not allowed to use a calculator to determine the sum. However you are welcome to write the numbers down and make use of the provided scratch paper. You submit an answer by clicking the OK button with your mouse. When you enter an answer the computer will immediately tell you whether your answer is correct or not. If Task 1 is the one randomly selected for your profit, then you get 1 dollar per problem you solve correctly in the 5 minutes. Your profit does not decrease if you provide an incorrect answer to a problem. We refer to this task as the Piece Rate task. If you have any questions before we begin, please press the help button or raise your hand.

OK

…

OK

The time is up.

OK

Please describe your feelings at this moment. [Four 1-10 scales with agree/disagree for feeling happy, angry, stressed and excited respectively.]

OK

We now ask you to wait for ten minutes - you can see the time counting down in the upper right corner of your screen. Please remain in your seat until the experiment automatically continues. Do not use your phone, private computer, etc, and do not communicate with any of the other players. OK

OK

We now ask you to provide the second saliva sample. The instructions are the same as before. If you have any questions, please raise your hand or press the help button.

OK

Task 2 – new rules! As in Task 1 you will be given 5 minutes to calculate the correct sum of a series of five 2-digit numbers. However for Task 2 your payment depends on your performance relative to that of a group of other participants who are here right now. Each group consists of four randomly grouped people. If Task 2 is the one randomly selected for payment, then your profit depends on the number of problems you solve compared to the three other people in your group. The individual who solves the most problems correctly will receive 4 dollars for every problem he or she solved correctly, while the other participants receive no profit. If there is a tie the payment will be split between the winners. We refer to this as the Tournament Task. You will not be informed of how you did in the tournament until the end of the experiment. If you have any questions before we begin, please press the help button or raise your hand.

OK

The time is up.

OK

Please describe your feelings at this moment. [Four 1-10 scales with agree/disagree for feeling happy, angry, stressed and excited respectively.]

OK

[Only showed in Experiment 2] You will now put the hand on which you do not have electrodes into a cooler with water. Please make sure that your hand is completely immersed in the water. Keep your hand in the water for 75 seconds. Some people experience some discomfort from having the hand in the water. If this happens to you, please remember that it is perfectly safe to have the hand in the water for 75 seconds (and for longer than that as well). Please try as hard as you can to keep the hand in the water for 75 seconds. In order to know when the 75 seconds have passed, please refer to the clock (counting up, in seconds) on your screen. After the 75 seconds have passed, please remove your hand and dry it off on the towel on your desk. Thereafter press the OK button.

OK

We now ask you to wait for 10 minutes [15 minutes in Experiment 2] - you can see the time counting down in the upper right corner of your screen. Please remain in your seat until the experiment automatically continues. Do not use your phone, private computer, etc, and do not communicate with any of the other players. OK

OK

We now ask you to provide the third saliva sample. The instructions are the same as before. If you have any questions, please raise your hand or press the help button.

OK

Task 3 – new rules! As in the previous two tasks you will be given 5 minutes to calculate the correct sum of a series of five 2-digit numbers. However you will now get to choose which of the two previous payment schemes you prefer to apply to your performance on the third task. If Task 3 is the one randomly selected for profit, then your earnings for this task are determined as follows. If you choose the Piece Rate, you receive 1 dollar per problem you solve correctly. If you choose the Tournament Rate, your performance will be evaluated relative to the performance of the other three participants of your group in the Task 2-tournament. If you correctly solve more problems than they did during Task 2, then you receive four times the profit from the piece rate, which means you will get 4 dollars per problem you solve correctly. You will receive no earnings for this task if you choose the tournament and do not solve more problems correctly now, than the others in your group did during Task 2. The next screen will ask you to choose whether you want the piece rate or the tournament rate applied to your performance in Task 3. You will then be given 5 minutes to calculate the correct sum of a series of five randomly chosen two-digit numbers in the same way as before. If you have any questions before we begin, please press the help button or raise your hand.

OK

Please decide which compensation scheme you prefer for Task 3.

OK

The time is up.

OK

Now we would like you to guess which your rank was, compared to the other members in your group. Please enter a number between 1 (meaning that you were the best in your group of four) and 4 (meaning that you were the last in your group of four). For each correct guess 2 dollars will be added to your profit from the experiment.

OK

…

OK

You can now see your earnings from the experiment.

OK

While we prepare your payments it would be great if you could answer a few questions.

OK
